# Nursing Students' Perspectives on Academic Dishonesty During Examinations and Assignments: A Cross-Sectional Study

**DOI:** 10.7759/cureus.52418

**Published:** 2024-01-17

**Authors:** Abdullah O Alotaibi, Jazi S Alotaibi, Wdad Alanazy, Mohammed Alqahtani, Gopal Nambi, Mohammad Shaphe, Mohammad Miraj, Faizan Kashoo

**Affiliations:** 1 Department of Nursing, College of Applied Medical Science, Majmaah University, Almajmaah, SAU; 2 Nursing Sciences and Public Health, Department of Biomedicine and Prevention, University of Rome Tor Vergata, Rome, ITA; 3 Department of Nursing, King Faisal University, Riyadh, SAU; 4 Department of Physical Therapy, Prince Sattam Bin Abdul Aziz University, Al Karj, SAU; 5 Department of Physical Therapy, Jazan University, Jazan, SAU; 6 Department of Physical Therapy and Health Rehabilitation, Majmaah University, Majmaah, SAU; 7 Department of Physical Therapy and Health Rehabilitation, Majmaah University, Riyadh, SAU

**Keywords:** cheating behavior, academic ethics, cross-sectional study, nursing students, academic cheating, academic integrity, academic dishonesty

## Abstract

Background and objective

Academic dishonesty or misconduct among nursing students is a crucial concern within educational institutions. In nursing education, academic dishonesty hinders the development of knowledge and skills among students, thereby jeopardizing both patient safety and the effectiveness of clinical practice. This study aimed to assess the prevalence and forms of academic dishonesty among nursing students in Saudi Arabia.

Methodology

The participants comprised 482 nursing students from two governmental universities in Saudi Arabia. A newly developed self-reported questionnaire was used to collect data on academic dishonesty, comprising two distinct sections: a 16-item Academic Dishonesty Questionnaire during examinations and an 11-item Academic Dishonesty Questionnaire related to assignments.

Results

A one-sample binomial test indicates a significant proportion of students engaging in at least one form of academic dishonesty (*n* = 452, 93.8%; *χ*²(1) = 19.176; *P* < 0.001). Notably, 432 (89.9%) students reported dishonesty in examinations and 385 (79.9%) in assignments. Multiple-response analysis of 7,712 responses from 482 students using the 16-item Examination Dishonesty Questionnaire showed that the majority of the students (*n* = 4,010, 52%) were cheating on the examination. Similarly, of the 5,302 total responses from the 11-item Assignment Dishonesty Questionnaire, 2,773 (52.3%) responses revealed engagement in academic dishonesty during the assignment. The most prevalent and statistically significant form of academic dishonesty during examinations was studying previous examination questions without the knowledge of the teacher (*n* = 370, 76.5%), followed by providing and collecting previous examination question papers (*n* = 316, 65.6%) and (*n* = 304, 63.1%), respectively. Similarly, the common and significant form of academic dishonesty during the completion of assignments included unfair collaboration (*n* = 331, 68.7%), allowing a friend to copy assignments (*n* = 304, 63.1%), and copying from the internet (*n* = 286, 59.3%) without citing the source (*P* < 0.001).

Conclusions

Our study identified a significant prevalence of academic dishonesty among Saudi nursing students, a particularly noteworthy concern within the context of a respected governmental educational institution. This emphasizes the need for implementing rigorous preventive measures to curb academic dishonesty. Based on the findings of our study, recommended interventions include providing educational workshops or similar initiatives to educate students on the consequences of cheating and plagiarism, using diverse questions to assess knowledge and skills during theory examinations and assignments, enforcing stringent penalties for copying and cheating, establishing a code of ethics, and proactively promoting ethical practices among nursing students by leveraging the influence of Islamic religious principles to address this issue.

## Introduction

Academic dishonesty, academic fraud, and misconduct are all unethical practices that undermine the integrity of the academic setting [1]. These terms share similar connotations and have gained attention, particularly in the context of dishonesty [2], cheating [3], and falsification [4] among students enrolled in various disciplines at universities [5]. The field of nursing education is not exempt, despite the implementation of diverse preventive measures [6-8]. The detrimental impact of such inappropriate conduct substantially undermines the acquisition of basic skills and knowledge among aspiring nursing professionals, consequently compromising the overall standard of healthcare [9].

Previous research has documented a substantial occurrence of academic dishonesty within nursing schools on a global scale [10]. A longitudinal study conducted in Italy revealed that a significant proportion of nursing students perceived that cheating behavior was acceptable and normal [11]. A study performed in the United States among a sample of 336 nursing students showed that more than half of the participants engaged in academic dishonesty [2]. A study conducted in Turkey involving a sample of 499 students pursuing degrees in midwifery, nursing, and nutrition-dietetics found that 43.9% of participants admitted to engaging in various forms of academic dishonesty during examinations [12]. A cross-sectional study among 361 nursing students from across Australia revealed that a significant proportion of participants engaged in acts of plagiarism [13]. The results also showed that the most common type of academic misconduct seen was intentionally lying about patients' vital signs and breaking client confidentiality without permission [13]. The widespread occurrence of academic dishonesty among nursing students might impact the well-being of patients entrusted to their care in the future. Trust plays a pivotal role in the relationship between nurses and patients, serving as an indicator of nurses’ competence, adherence to professional ethics, and integrity. Accordingly, the prevalence of academic dishonesty in nursing education is a crucial issue that raises questions regarding the integrity and professionalism of nurses, thus warranting substantial attention within the nursing field.

A study reported that in Saudi Arabia and Egypt, academic misconduct was influenced by various individual sociodemographic and cultural factors [14], including gender, pressure from parents to obtain higher grades, and a lack of knowledge about different forms of academic dishonesty. Another study showed that nursing students may not categorize all forms of academically dishonest behavior as inherently dishonest [15]. The findings further indicated that a positive correlation existed between individuals’ perceptions of behaviors observed in the classroom and those observed in the clinical setting [15]. In research conducted in Saudi Arabia, there was a significant prevalence of dishonest behavior among medical students [16]. The study also found that male students were more inclined to admit to engaging in academic dishonesty than their female peers [16].

The sociocultural background, religious beliefs, ethical values, and attitudes of individuals residing in the Middle East, particularly Saudi Arabia, exhibit notable distinctions from those of individuals residing in other regions [17,18]. A study conducted in Saudi Arabia and Egypt showed that academic misconduct was influenced by various individual sociodemographic and cultural factors [14]. Furthermore, the increasing need for nurses necessitates the acquisition of up-to-date knowledge, skills, and advanced techniques to enhance the quality of patient care both nationally and globally [19]. The demand for professional nursing care is prevalent not only in Saudi Arabia but also in other countries worldwide [20]. Saudi Arabia continues to face challenges in effectively integrating Saudi nationals into the nursing profession. To address such challenges, Saudi Arabia is making substantial investments in the development of nursing education, to improve the quality of clinical care provided to patients [21]. The field of nursing in the country has markedly progressed in terms of both educational advancements and clinical practices [22]. Hence, the prevalence of academic dishonesty among prospective nurses could have a substantial influence on the achievement of professionalism, diligence, and efficiency, thereby affecting the overall standard of healthcare quality in Saudi Arabia.

This study aimed to identify the most common forms of academic misconduct during examinations and the completion of academic assignments among nursing students in Saudi Arabia. To the best of our knowledge, this study represents the first attempt to identify the prevalent forms of academic dishonesty during examinations and the completion of assignments in the country. The identification of different forms of academic dishonesty is crucial in guiding academic institutions in establishing policies and implementing measures to address and prevent such unethical conduct among nursing students.

## Materials and methods

Design

This study adopted a cross-sectional design to determine the prevalence of academic dishonesty among nursing students from two different universities in Saudi Arabia. The participants from these two major universities in the central region of Saudi Arabia could serve as a close representation of the nationwide scenario.

Sampling and setting

A total of 866 nursing students from two governmental universities who met the inclusion criteria were invited to participate in the study. Of them, 482 students completed the survey questionnaire, yielding a response rate of 55.65%. Students in their preparatory, second, third, or fourth year of their bachelor’s degree were included, while internship nursing students were excluded.

Sample size calculation

The sample size for this study was determined using the online Raisoft sample size calculator. It was based on a response rate of 50%, a 95% confidence interval, and a margin of error of 5%. Ultimately, a sample size of 477 participants was deemed necessary.

Procedure

The study was approved by the ethical committee at the governmental university under approval number 53/4014 in August 2022. The study adhered to the principles outlined in the Helsinki Declaration. The data were collected using Google Surveys, an internet-based platform. After the necessary authorization was obtained from the appropriate university authorities, a survey hyperlink was distributed to the email addresses of eligible students. This hyperlink was accompanied by an introductory information document that provided a clear explanation of the study’s objectives and methodology using easily understandable language. The study adopted a voluntary participation approach, ensuring participant confidentiality and refraining from offering any incentives. The participants were appropriately informed of their right to withdraw from the study at any time and were provided with the relevant contact details of the principal investigator for any questions or concerns. The collected data were handled with care to maintain confidentiality, and the results were presented in a condensed format, safeguarding the privacy of individual participants by avoiding the disclosure of their identities and personal information. Following the initial approval in August 2022, a complete dataset of 482 participants was obtained and analyzed after February 2023.

Instrument: questionnaire development and reliability

The steps outlined by Chalhoub-Deville [23] were employed in the development of the Academic Dishonesty Questionnaire (ADQ), employing a systematic approach to ensure the reliability and validity of the measurement instruments.

Step 1: Determining a Theoretical Construct and Items Pooled From Previously Published Researches

**Figure 1:**
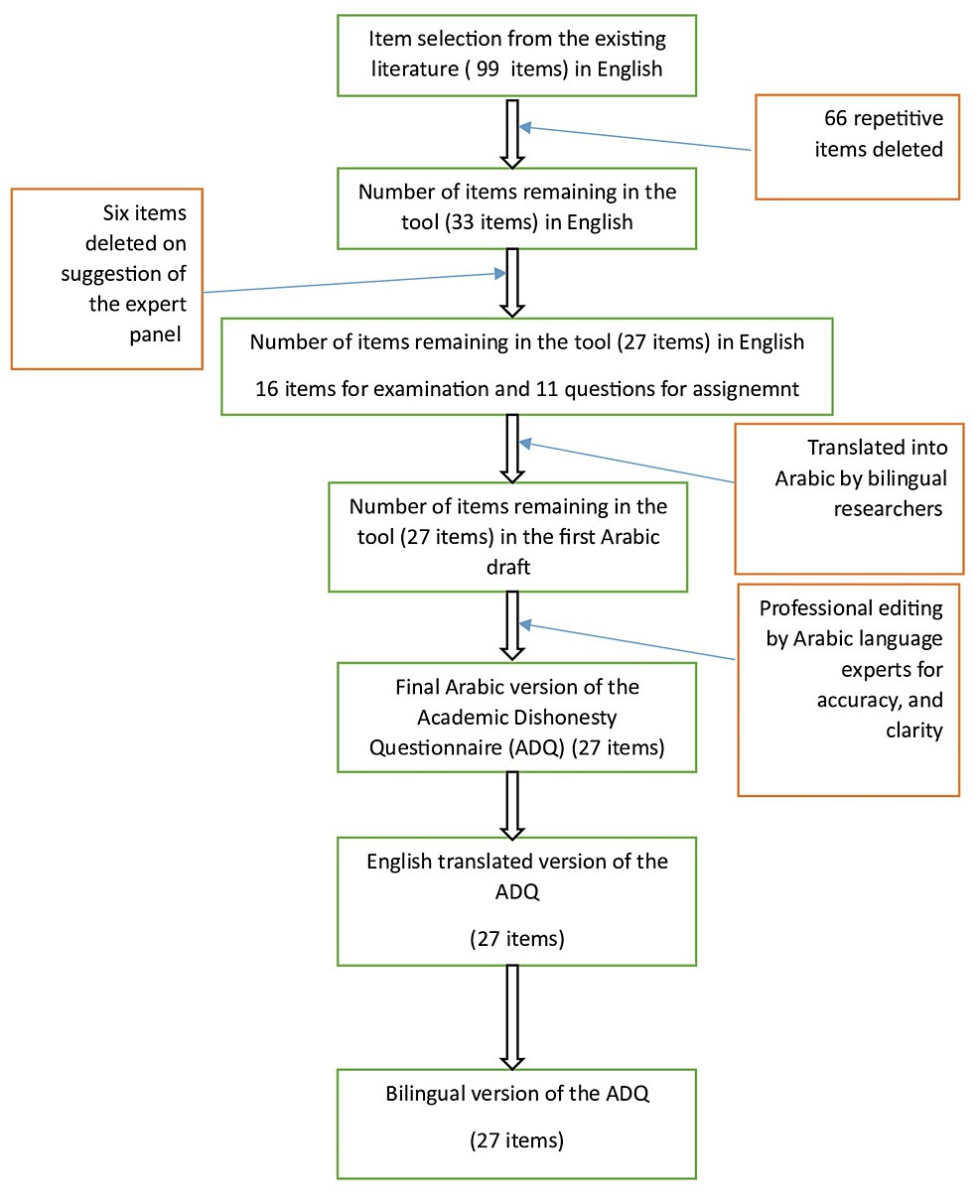
Flowchart of the steps followed to develop the questionnaire. Image credit: Abdullah M. Alotaibi. ADQ, Academic Dishonesty Questionnaire

Relevant items measuring a similar construct were derived from the previously published research [2,10,24-27]. The findings of these previous studies were thoroughly examined to identify suitable components for the questionnaire, which aimed to evaluate the prevalence of academic dishonesty during examination and completion of assignments among nursing students. Figure 1 depicts the flowchart illustrating the process of tool development. At the outset, a set of 99 items was identified. Redundant questions were eliminated from the original set of items, resulting in a final set of 20 questions on academic dishonesty during the examination and 13 questions on academic dishonesty during the completion of assignments.

Step 2: Expert Review and Content Validity

The questions were submitted to an external panel consisting of five reviewers with considerable expertise in the field of nursing education in Saudi Arabia to assess the content validity of the questionnaire. The instrument was evaluated by the external panel by the criteria for achieving content validity. Based on the recommendations put forth by the expert reviewers, six questions were removed, resulting in a revised total of 27 questions. Furthermore, certain items were modified slightly based on the suggestions provided. The instrument developed in this study was ultimately called ADQ.

Step 3: Translation Into Arabic

To ensure validity and reliability across diverse Arabic cultural and linguistic settings, Beaton's recommendations [28] were employed for the translation of the ADQ. The methodology comprised the following essential steps: Initial translation of the English version of the 27-item questionnaire to Arabic by the first three authors (bilingual) of this article, followed by a comprehensive review to identify potential cultural and linguistic barriers by the fourth and fifth authors. Expert review: Bilingual professionals, including an English language expert, a computer professional, and three nursing subject experts, utilized their expertise in both languages to guarantee the accuracy of the questionnaire translation from English to Arabic. The experts preserved the intended meaning of the questions while considering the cultural context for relevance and addressing any discrepancies. The questionnaire was back-translated to English to identify discrepancies. A final Arabic version was piloted among 10 nursing students, and feedback from the pilot study was compiled by an independent expert, with any discrepancies resolved through mutual consensus among experts (Figure 1).

Step 4: Test-Retest Reliability and Internal Consistency of the Dual-Language Questionnaire

A test-retest study was conducted among a sample of 50 nursing students to assess the intra-rater reliability of the ADQ. These students were not informed in advance that they would be asked to complete the questionnaire twice. The study involved a 14-day interval between administrations of the questionnaire. Participants were requested to fill out the questionnaire and offer feedback on its clarity in both English and Arabic dialects. The correlation coefficient between the test and retest scores of the questionnaire was 0.96, with a confidence interval of 0.93-0.98, indicating a strong positive relationship. The intra-class correlation coefficient was 0.977, with a confidence interval of 0.974-0.988, indicating a high degree of agreement among the participants’ responses on the ADQ. This high ICC value demonstrates the robust agreement among the individuals who completed the ADQ, suggesting that the scale reliably measures the construct it was designed to assess. The narrow confidence interval further bolsters the credibility of the findings, indicating the precision and consistency of the ICC estimate.

Step 5: Factor Analysis of Questionnaire (Construct Validity)

The robustness of the construct validity of the instrument was further evaluated with principal factor analysis after obtaining the data from 482 nursing students. Principal component analysis (PCA) was employed as the extraction method, and Varimax rotation was utilized to identify noncorrelated factors in evaluating the factor structure of the 27-item questionnaire designed to assess academic dishonesty among nursing students. The dataset consisted of 16 items related to examination and 11 items related to assignment dishonesty. We utilized the Kaiser-Meyer-Olkin (KMO) measure to verify the adequacy of the sample size, and Bartlett's test of sphericity confirmed the factorability of the data. The analysis revealed a one-factor solution that accounted for 80.5% of the total variance. The one-factor structure implies that academic dishonesty among nursing students, as measured by this questionnaire, is a homogeneous construct. The unidimensional nature of this questionnaire suggests that academic dishonesty among nursing students is a cohesive concept.

The final version of the questionnaire

The final questionnaire was introduced in dual language with dichotomous answers to 27 items. The final version of the questionnaire is attached as supplementary data with this paper (Appendix).

Statistical analysis

The survey questionnaire was gathered from all participants, and the responses were evaluated. The data were analyzed and summarized for review. All statistical analyses were conducted using IBM SPSS Statistics for Windows, Version 20.0 (IBM Corp., Armonk, NY). Descriptive statistics (i.e., percentages and frequencies) were used to present the data, and a binomial and multinomial regression analysis was conducted to assess the relationship between the variables. Binomial tests were used to assess the difference between the responses for each item in the questionnaire. Several psychometric tests were conducted to test the reliability and validity of the newly developed questionnaire. A *P*-value of <0.05 was considered statistically significant.

## Results

Of the 866 eligible nursing students, 482 participated in the study, yielding a response rate of 55.6%. Among the 482 nursing students, 293 (60.8%) were from University A, while 189 (39.2%) were from University B. The majority of the nursing students (*n* = 277, 57.5%) were aged from 21 to 25 years, while only 1 (2.3%) was aged over 30 years. The majority of the nursing students were single (*n* = 453, 94%) and women (*n* = 285, 59.1%). The number of nursing students across the four academic years was almost equal. The demographic data of the nursing students are summarized in Table 1.

**Table 1:** Demographic data. ^*^One-sample binomial test (*P *< 0.001). OR, odds ratio; CI, confidence interval

Characteristics	Variables	Participants, *n* (%)	Ever cheated, *n* (%)	Never cheated, *n *(%)	OR (95% CI)	*P*-value
Gender	Male	195 (40.5)	185 (94.9)	10 (5.1)	1.3 (0.6-3.0)	0.413
	Female	287 (59.5)	267 (93)	20 (7)	1	-
Age group (years)	15-20	175 (36.3)	164 (93.7)	11 (6.3)	1.4 (0.1-12.7)	0.715
	21-25	277 (57.5)	259 (93.5)	18 (6.5)	1.4 (0.1-11.8)	0.735
	26-30	19 (3.9)	19 (100)	0 (0)	1.6 (0.5-2.7)	0.998
	>30	11 (2.3)	10 (90.9)	1 (9.1)	1	-
Marital status	Single	453 (94)	424 (93.6)	29 (6.4)	1.9 (0.2-14.5)	0.530
	Married	29 (6)	28 (96.6)	1 (3.4)	1	
Academic year	Preparatory year	115 (23.9)	113 (98.3)	2 (1.7)	1.9 (0.3-11.0)	0.435
	Second year	132 (27.4)	113 (85.6)	19 (14.4)	0.2 (0.06-0.6)	0.006
	Third year	117 (24.3)	112 (95.7)	5 (4.3)	0.7 (0.2-3.0)	0.725
	Fourth year	118 (24.5)	114 (96.6)	4 (3.4)	1	-
University	University A	293	266 (90.8)	27 (9.2)	0.1 (0.04-0.5)	0.003
	University B	189	186 (98.4)	3 (1.6)	1	-
	Total	482	452 (93.7)	30 (6.3)	-	0.001*

Of the 482 nursing students, approximately 30 (6.2%) reported that they were never involved in any form of cheating. In the analysis of the demographic variables, it was observed that the second-year nursing students exhibited a lower propensity for engaging in academic dishonesty (*n *= 19, 14.4%; odds ratio [OR] = 0.2) than did the preparatory-year (*n *= 2, 1.7%), third-year (*n *= 5, 4.3%), and fourth-year (*n* =4, 3.4%) nursing students. These findings demonstrated statistical significance (*P* = 0.006). The prevalence of academic dishonesty among the nursing students at University A (*n* = 27, 9.2%; OR = 0.1) was significantly lower than that among the nursing students at University B (*n* = 3, 1.6%) (*P* = 0.003). The results of a one-sample binomial test revealed a significant proportion of students engaging in at least one form of academic dishonesty (*n* = 452, 93.7%; *χ*²(1) = 19.176; *P* < 0.001).

Cheating during examinations

Table 2 presents the responses to 16 questions related to the act of cheating during examinations. The overall prevalence of cheating during the examination was 4,010 (52.0%). A significant (*P* < 0.001) number of nursing students reported engaging in various forms of cheating, such as studying previous materials without their teacher’s knowledge (*n* = 370, 76.5%), obtaining previous years’ questions from different sources (*n* = 363, 75.2%), following the lead of senior students who had already taken the examination (*n* = 316, 65.6%), and participating in group activities to collect questions from previous examinations (*n* = 304, 63.1%). On the contrary, the majority of students reported not going to the restroom during the examination to cheat (*n* = 265, 55%), exchanging papers with other students (*n* = 274, 56.8%), taking the examinations for other students (*n* = 278, 57.7%), or asking others to take their examination (*n* = 279, 57.9%). An analysis of multiple responses from 16-item questionnaires on examinations from 482 students resulted in 7,712 responses. Within this dataset, a nonsignificant proposition of students (4,010, 52%) was found to be involved in cheating during examinations (*P* = 0.595; *Z* = -0.53).

**Table 2:** Cheating/academic dishonesty in the examination. ^*^One sample Binomial test.

S. no.	Statement	Never cheated, *n* (%)	Cheated, *n* (%)	*P*-value*
1	I studied exam questions from past exams without the instructor’s knowledge.	112 (23.2)	370 (76.5)	0.001*
2	I obtained questions and/or answers from others who had already taken the exam.	119 (24.7)	363 (75.2)	0.001*
3	I provided exam questions to other students who had to take the same exam later.	166 (34.4)	316 (65.6)	0.001*
4	I participated in collecting exam questions as a group for other students.	178 (36.9)	304 (63.1)	0.001*
5	I copied from other students during an exam with their knowledge.	237 (49.2)	245 (50.9)	0.75
6	I sought verbal information from other students during an exam.	248 (51.5)	234 (48.5)	0.554
7	I copied from other students during an exam without their knowledge.	252 (52.3)	230 (47.7)	0.339
8	During examinations, I used non-verbal signals to send/receive answers to/from my friends.	253 (52.5)	229 (47.6)	0.295
9	I wrote notes on tables, walls, or other equipment during exams.	259 (53.7)	223 (46.3)	0.111
10	I used dishonest means to obtain information about the content of exams before they were administered.	259 (53.7)	223 (46.3)	0.111
11	I sat near proficient students to improve my chances of cheating successfully.	260 (53.9)	222 (46.0)	0.092
12	I used prohibited aids (e.g., hidden notes, calculators, and other devices) during examinations.	263(54.6)	219 (45.4)	0.523
13	I obtained information by leaving the examination room (e.g., going to the restroom).	265 (55.0)	217 (45.0)	0.032*
14	I exchanged examination papers with other students.	274 (56.8)	208 (43.0)	0.003*
15	I took an exam for another student.	278 (57.7)	204 (42.2)	0.001*
16	I asked others to take exams for me.	279 (57.9)	203 (42.2)	0.001*
	Total prevalence	3,702 (48.0)	4,010 (52.0)	0.595*

Cheating during the completion of assignments

There were 11 questions related to cheating during completion of assignments (Table 3). The overall prevalence of cheating during completion of assignments was 2,529 (47.7%). Statistically significant (*P* < 0.001) number of nursing students reported that they often sought help from others (*n* = 331, 68.7%) even when their teacher had instructed them to complete the assignment by themselves, allowed their friends to copy assignments (*n* = 304, 63.1%), and paraphrased materials without citing sources (*n* = 286, 59.3%). On the contrary, a significant number of students reported never involved in the fabrication or falsification of a bibliography (*n* = 265, 55%). Multiple-response analysis from an 11-item questionnaire for assignments from 482 students yielded 5,302 responses, out of which a significant proportion of responses (*n* = 2773, 53.3%) indicated students cheating while completing their assignments (*P* < 0.001; *Z* = 5.456).

**Table 3:** Cheating/academic dishonesty in the assignment or homework. ^*^One-sample Binomial test.

S. no.	Questions	Never cheated, *n* (%)	Cheated, *n *(%)	*P*-value*
1	I collaborated on assignments when the instructor asked for individual work.	151 (31.3)	331 (68.7)	0.001*
2	I allowed friends to copy my homework assignments.	178 (36.9)	304 (63.1)	0.001*
3	I copied or paraphrased material from internet sources, books, magazines, or journals without citing them in my papers.	196 (40.7)	286 (59.3)	0.001*
4	I asked others to do my assignments.	236 (49)	246 (51)	0.682
5	I provided false excuses to gain extra time on projects/assignments from instructors.	237 (49.2)	245 (50.8)	0.751
6	I did assignments for other students.	244 (50.6)	238 (49.4)	0.823
7	I presented other students’ ideas as my own in my papers.	251 (52.1)	231 (47.9)	0.387
8	I resubmitted papers of my own that I had already submitted in another course without the instructor’s knowledge.	254 (52.7)	228 (47.3)	0.255
9	I hired others to do my work and submit it as my own.	255 (52.9)	227 (47.1)	0.219
10	I submitted assignments in my own name after having them prepared by others.	262 (54.4)	220 (45.6)	0.062
11	I fabricated or falsified a bibliography.	265 (55)	217 (45)	0.032*
	Total	2,529 (47.7)	2,773 (47.7)	0.001*

## Discussion

To our knowledge, this study is the first to evaluate the crucial and sensitive issue of academic dishonesty among nursing students at two major institutions in Saudi Arabia. The current investigation unveils a notable prevalence of academic dishonesty among nursing students in Saudi Arabia in various forms during examinations and the completion of assignments. Based on the findings, it is recommended that educational authorities in Saudi Arabia formulate strategic plans and policies aimed at curbing unethical behavior among nursing students. These measures may include implementing moral and ethical educational programs, establishing disciplinary measures, providing teacher training on addressing contemporary instances of academic dishonesty during examinations and the completion of assignments, and ensuring the availability of counseling services.

This study found that 452 (93.7%) nursing students reported being involved in at least one form of academic dishonesty, with 432 (89.9%) reporting dishonesty in examinations and 385 (79.9%) while completing their assignments. Academic dishonesty has been found to have adverse effects on both the integrity of the workplace and the overall quality of the healthcare system [9]. This study is noteworthy for being conducted in Saudi Arabia, a country recognized for its conservative and religious values. The adherence to Islamic Shari’ah law in this context promotes and underscores the significance of honesty and ethical conduct.

This study showed that there was no significant disparity in academic dishonesty between male and female nursing students. In contrast, a previous study reported that male nursing students exhibited a higher tendency to engage in academic dishonesty than did their female counterparts [2]. This disparity in the results could be attributed to the unequal number of participants in the study. Female students may exhibit a greater degree of concern regarding the potential detection of cheating and the subsequent social stigma that may ensue. In contrast, male students tend to display a more nonchalant, careless, or audacious attitude toward academic dishonesty, seemingly unaffected by the potential repercussions that may arise in the event of being caught. On the contrary, a study reported that a significant number of female students exhibited dishonesty in clinical settings [26]. The observed discrepancy in academic dishonesty between genders may be attributed to the increased likelihood of detection during examinations compared with clinical placements.

Academic dishonesty during examination

During examination more than three-fourths of the students reported engaging in some form of academic dishonesty during or before examination. The most prevalent form examination dishonesty was studying previous question paper without the knowledge of instructor, collecting questions of previous examination question papers. Likewise, a study conducted in the United States with 973 participants revealed that 96% acknowledged engagement in at least one form of unethical conduct out of a pool of 21 distinct behaviors observed in both classroom and clinical environments [15]. Furthermore, a significant proportion of 60% admitted to engaging in five or more of these dishonest behaviors [15]. Another study conducted in the United States reported that a significant proportion of nursing students (65% in the classroom setting and 54% in the clinical setting) participated in acts of dishonesty [2]. Furthermore, nearly half of the participants admitted to engaging in three or more forms of dishonest behavior in both classroom and clinical settings [2]. On a positive note, more than half of the students in our study reported abstaining from certain dishonest acts during exams, such as cheating during restroom breaks, or exchanging question papers or attempting to have someone else take their examination. The total number of multiple responses (*n *= 7,712) from 482 participants on the 16-item examination dishonesty questionnaire revealed that more than half were engaged in academic dishonesty. However, when compared to non-cheaters, the reported engagement in academic dishonesty during examinations did not reach statistical significance. However, it is crucial to interpret these findings with caution, as the statistical test employed used a criterion of a 50% proposition, which may not align with the more commonly accepted academic standards, where the acceptable percentage for academic dishonesty must be close to zero. This emphasizes the urgency for academic institutions to effectively address and prevent academic dishonesty.

Academic dishonesty during completion of assignments

Our study found that close to three-fourths of the sample of students were involved in asking for help from other students, copying others' assignments, and engaging in plagiarism. Similarly, a study conducted among 499 science students in Turkey revealed that only 49.1% of participants acknowledged citing sources during completion of their assignments [12]. In a study conducted in Australia, students demonstrated attempts to mitigate plagiarism; however, their efforts failed owing to a lack of interest, time constraints resulting from conflicting obligations, and inadequate comprehension of the academic integrity policy [27]. Saudi Arabia lacks a sufficient number of exploratory studies aimed at identifying the underlying factors contributing to the high prevalence of plagiarism among nursing students. The total number of multiple responses (*n *= 5,302) from 482 participants on the 11-item assignment dishonesty questionnaire revealed that more than half were engaged in academic dishonesty. Additionally, when compared to non-cheaters, the reported engagement in academic dishonesty during assignments reached statistical significance. There is a pressing need to bring the current menace of academic dishonesty among students. Any form of dishonesty is unequivocally unacceptable, particularly in disciplines such as healthcare, where integrity and ethical conduct form the bedrock of professional competence.

Strategies to prevent academic dishonesty

The issue of academic misconduct necessitates a serious approach. After implementation of punitive measures for a considerable period, it may be appropriate to consider enhancing the intrinsic motivation and ethical self-perception associated with upholding academic integrity. To proactively address the issue, several studies have proposed that it is necessary to provide students with knowledge and instruction to deter instances of copying during examination. Similarly, educators globally have been suggested to confront the issue of academic dishonesty within their educational establishments by undertaking comprehensive investigations into strategies that prove to be effective in mitigating its occurrence. Based on empirical evidence, it is imperative that students possess a comprehensive understanding of the ramifications of engaging in academic dishonesty, particularly in relation to their prospective professional trajectories and the overall caliber of healthcare services they will deliver to their local communities or country or the global population. It is essential to conduct a thorough evaluation and revision of professionalism- and medical ethics-related courses within nursing degree programs. These courses, introduced as remedial measures, aim to enhance the effectiveness of behavioral change. Moreover, religious inclination and adherence may serve as a deterrent against academic misconduct, given that religious beliefs exert substantial influence over the actions, thoughts, mental states, and emotional experiences of individuals. Saudi Arabia is widely recognized as one of the most devout states globally, known for its strict implementation of Islamic Shari’ah law. Given the strong religious convictions prevalent in the country, there exists an opportunity to harness the power of Islamic beliefs to raise awareness and discourage academic dishonesty among students.

Limitations

The inherent nature of a cross-sectional study introduces potential inaccuracies due to reliance on self-reporting, which can be susceptible to influences such as social desirability bias or limited self-awareness. The study participants were exclusively sourced from two governmental universities, leading to a lack of heterogeneity. In a comparable manner, owing to the self-reported nature of the data, there exists the potential for an elevated proportion of students participating in academically dishonest behaviors. Notwithstanding the noteworthy discoveries derived from this investigation, it is imperative to acknowledge the constraints inherent in the study. The study encountered several types of biases, including response bias and observer effects. Moreover, the analysis did not adequately account for confounding variables, such as the disproportionate representation of men among students living away from home. This factor could influence the impact of residential location on the outcomes. Additionally, we intended to investigate the behaviors exhibited by noncompliant students and explore the underlying rationale behind their actions.

## Conclusions

Our study identified a significant prevalence of academic dishonesty among Saudi nursing students, a particularly noteworthy concern within the context of a respected governmental educational institution. This emphasizes the need for implementing rigorous preventive measures to curb academic dishonesty. Based on the findings of our study, recommended interventions include providing educational workshops or similar initiatives to educate students on the consequences of cheating and plagiarism, using diverse questions to assess knowledge and skills during theory examinations and assignments, enforcing stringent penalties for copying and cheating, establishing a code of ethics, and proactively promoting ethical practices among nursing students by leveraging the influence of Islamic religious principles to address this issue.

## Appendices

**Table 4:** Questionnaire.

Academic Dishonesty Questionnaire (ADQ) English-Arabic Version ستبيان خيانة الأمانة الأكاديمية النسخة الإنجليزية والعربية			
Instructions تعليمات			
Please answer the following question with a yes or no answer. Circle or underline the answer. يرجى الإجابة على السؤال التالي بإجابة نعم أو لا. ضع دائرة حول الإجابة أو تسطيرها.			
Your responses and identity will remain anonymous. ستبقى ردودك وهويتك مجهولة.			
	Section 1: Questionnaire related to academic dishonesty in Examination (16 –items) الأسئلة المتعلقة بالامتحان (16 مادة)		
	I studied exam questions from past exams without the instructor’s knowledge. قمت بدراسة أسئلة الامتحانات من الامتحانات السابقة دون علم المعلم.	Yes/no نعم / لا	
	I provided exam questions to other students who had to take the same exam later. قدمت أسئلة الامتحان للطلاب الآخرين الذين اضطروا لإجراء نفس الاختبار لاحقًا.	Yes/no نعم / لا	
	I obtained questions and/or answers from others who had already taken the exam. حصلت على أسئلة و/ أو إجابات من الآخرين الذين سبق لهم إجراء الإمتحان	Yes/no نعم / لا	
	I participated in collecting exam questions as a group for other students. شاركت في جمع أسئلة الامتحان كمجموعة لطلاب آخرين.	Yes/no نعم / لا	
	I allowed copying my answers for other students during an exam with my knowledge. سمحت للآخرين بنقل إجاباتي أثناء الإمتحان.	Yes/no نعم / لا	
	I copied the answers from other students during an exam without their knowledge. بنقل الاجابات من طلاب آخرين أثناء الامتحان بعلمهم. لقد قمت	Yes/no نعم / لا	
	I asked others to take exams for me. ا طلبت من الآخرين الجلوس لأداء الامتحان بدلًا عني	Yes/no نعم / لا	
	I took an exam for another student. قمت بإجراء الامتحان بدلًا عن طالب آخر.	Yes/no نعم / لا	
	I wrote notes on tables, walls, or other equipment during exams. اكتبت ملاحظات على الطاولات أو الجدران أو غيرها من المعدات أثناء الامتحانات.	Yes/no نعم / لا	
	I sought verbal information from other students during an exam. ا طلبت معلومات شفهية من طلاب آخرين أثناء الامتحان.	Yes/no نعم / لا	
	I obtained information by leaving the examination room (e.g., going to the restroom). حصلت على المعلومات من خلال مغادرة غرفة الفحص/ الامتحان (على سبيل المثال: الذهاب إلى الحمام).	Yes/no نعم / لا	
	I exchanged examination papers with other students during the examination. تبادلت أوراق الامتحان مع طلاب آخرين.	Yes/no نعم / لا	
	During examinations, I used non- verbal signals to send/receive answers to/from my friends. استخدمت إشارات لإرسال/ تلقي إجابات من/ إلى أصدقائي أثناء الامتحانات.	Yes/no نعم / لا	
	I used prohibited aids (e.g., hidden notes, calculators, and other devices) during examinations. استخدمت وسائل مساعدة محظورة (على سبيل المثال: الملاحظات المخفية، والآلات الحاسبة، وغيرهامن الأجهزة) أثناء الامتحانات	Yes/no نعم / لا	
	I sat near proficient students to improve my chances of cheating successfully. الجلوس بالقرب من الطلاب الأكفاء لزيادة فرصي في الغش بنجاح.	Yes/no نعم / لا	
	I used dishonest means to obtain information about the content of exams before they were administered. استخدمت وسائل غير شريفة للحصول على معلومات حول محتوى الامتحانات قبل إجرائها	Yes/no نعم / لا	
	Section 2: Questionnaire related to dishonesty in Assignments الأسئلة المتعلقة بالمهمة		
	I copied some or all of another student’s paper. قمت بنسخ بعض أو كل أوراق طالب آخر.	Yes/no نعم / لا	
	I copied or paraphrased material from internet sources, books, magazines, or journals without citing them in my papers. نقل أو إعادة صياغة مواد من الإنترنت أو الكتب أو المجلات أو المذكرات دون ذِكر مصدرها كمرجع في أوراقي.	Yes/no نعم / لا	
	I collaborated on assignments when the instructor asked for individual work. التعاون لآداء الواجبات عندما طلب المحاضر العمل بصورة فردية.	Yes/no نعم / لا	
	I fabricated or falsified a bibliography. قمت بتزييف أو تزوير قائمة المراجع.	Yes/no نعم / لا	
	I resubmitted papers of my own that I had already submitted in another course without the instructor’s knowledge. اعادة تقديم المهمات (الاسيمنات) أو الواجبات الخاصة بي التي قدمتها بالفعل في مقرر آخر دون علم المحاضر	Yes/no نعم / لا	
	I did assignments for other students. قمت بالواجبات أو المهمات أو (الاسيمنات) الخاصة بالطلاب الآخرين	Yes/no نعم / لا	
	I presented other students’ ideas as my own in my papers. قدمت أفكار الطلاب الآخرين على أنها أفكاري في المهمات (الاسيمنات) الخاصة بي.	Yes/no نعم / لا	
	I allowed friends to copy my homework assignments. السماح للأصدقاء بنقل واجباتي أو مهماتي (اسيمنتاتي) المنزلية	Yes/no نعم / لا	
	I submitted assignments in my own name after having them prepared by others. سلمت الواجبات أو المهمات باسمي الخاص بعد أن أعدها الآخرون	Yes/no نعم / لا	
	I provided false excuses to gain extra time on projects/assignments from instructors. قدمت أعذار كاذبة لكسب وقت إضافي في المشاريع/ الواجبات من المحاضر.	Yes/no نعم / لا	
	I hired others to do my work and submit it as my own. توكيل آخرين للقيام بواجبي وتقديمه على أنه عملي.	Yes/no نعم / لا	

## References

[1] Maoz E, Gorbunov I, Danino E, Zerahia M (2022). An honest cheater: perception of self-concept, academic and clinical dishonesty among nursing students. Nurse Educ Today.

[2] Krueger L (2014). Academic dishonesty among nursing students. J Nurs Educ.

[3] Ebaid IE (2021). Cheating among accounting students in online exams during Covid-19 pandemic: exploratory evidence from Saudi Arabia. Asian J Econ Financ Manag.

[4] Anoopa KR, Mathews M, Greeshma P V, Thomas M, Ambika S (2019). Academic integrity among nursing students. Int J Heal Sci Res.

[5] Fadlalmola HA, Elhusein AM, Swamy DS, Hussein MK, Mamanao DM, Mohamedsalih WE (2022). Plagiarism among nursing students: a systematic review and meta-analysis. Int Nurs Rev.

[6] Palmer JL, Bultas M, Davis RL, Schmuke AD, Fender JB (2016). Nursing examinations: promotion of integrity and prevention of cheating. Nurse Educ.

[7] Dibartolo MC, Walsh CM (2010). Desperate times call for desperate measures: where are we in addressing academic dishonesty?. J Nurs Educ.

[8] Błachnio A, Cudo A, Kot P (2022). Cultural and psychological variables predicting academic dishonesty: a cross-sectional study in nine countries. Ethics Behav.

[9] Alnajjar PhD HA, Abou Hashish PhD EA (2021). Academic ethical awareness and moral sensitivity of undergraduate nursing students: assessment and influencing factors. SAGE Open Nurs.

[10] McCabe DL (2009). Academic dishonesty in nursing schools: an empirical investigation. J Nurs Educ.

[11] Macale L, Ghezzi V, Rocco G, Fida R, Vellone E, Alvaro R (2017). Academic dishonesty among Italian nursing students: a longitudinal study. Nurse Educ Today.

[12] Oran NT, Can HÖ, Şenol S, Hadımlı AP (2016). Academic dishonesty among health science school students. Nurs Ethics.

[13] Birks M, Smithson J, Antney J, Zhao L, Burkot C (2018). Exploring the paradox: a cross-sectional study of academic dishonesty among Australian nursing students. Nurse Educ Today.

[14] (2022). Farahat A: Elements of academic integrity in a cross-cultural middle eastern educational system: Saudi Arabia, Egypt, and Jordan case study. Int J Educ Integr.

[15] McClung EL, Schneider JK (2018). Dishonest behavior in the classroom and clinical setting: perceptions and engagement. J Nurs Educ.

[16] Abdulghani HM, Haque S, Almusalam YA (2018). Self-reported cheating among medical students: an alarming finding in a cross-sectional study from Saudi Arabia. PLoS One.

[17] Aldossary A, While A, Barriball L (2008). Health care and nursing in Saudi Arabia. Int Nurs Rev.

[18] Miller-Rosser K (2006). Historical, cultural, and contemporary influences on the status of women in nursing in Saudi Arabia. Online J Issues Nurs.

[19] AlYami MS, Watson R (2014). An overview of nursing in Saudi Arabia. J Heal Spec.

[20] Alamri M (2011). Higher education in Saudi Arabia. J High Educ Theory Pract.

[21] Alnowibet K, Abduljabbar A, Ahmad S (2021). Healthcare human resources: trends and demand in Saudi Arabia. Healthcare (Basel).

[22] Alsufyani AM, Alforihidi MA, Almalki KE, Aljuaid SM, Alamri AA, Alghamdi MS (2020). Linking the Saudi Arabian 2030 vision with nursing transformation in Saudi Arabia: roadmap for nursing policies and strategies. Int J Afr Nurs Sci.

[23] Chalhoub-Deville M (1997). Theoretical models, assessment frameworks and test construction. Lang Test.

[24] Park EJ, Park S, Jang IS (2013). Academic cheating among nursing students. Nurse Educ Today.

[25] Keçeci A, Bulduk S, Oruç D, Çelik S (2011). Academic dishonesty among nursing students: a descriptive study. Nurs Ethics.

[26] Abusafia AH, Roslan NS, Yusoff DM, Mat Nor MZ (2018). Snapshot of academic dishonesty among Malaysian nursing students: a single university experience. J Taibah Univ Med Sci.

[27] Mukasa J, Stokes L, Mukona DM (2023). Academic dishonesty by students of bioethics at a tertiary institution in Australia: an exploratory study.

[28] Beaton D, Bombardier C, Guillemin F, Ferraz MB (2002). Recommendations for the cross-cultural adaptation of health status measures. New York Am Acad Orthop Surg.

