# Correction: Nursing Students' Perspectives on Academic Dishonesty During Examinations and Assignments: A Cross-Sectional Study

**DOI:** 10.7759/cureus.c175

**Published:** 2024-05-10

**Authors:** Abdullah O Alotaibi, Jazi S Alotaibi, Wdad Alanazy, Mohammed Alqahtani, Gopal Nambi, Mohammad Shaphe, Mohammad Miraj, Faizan Kashoo

**Affiliations:** 1 Department of Nursing, College of Applied Medical Science, Majmaah University, Almajmaah, SAU; 2 Nursing Sciences and Public Health, Department of Biomedicine and Prevention, University of Rome Tor Vergata, Rome, ITA; 3 Department of Nursing, King Faisal University, Riyadh, SAU; 4 Department of Physical Therapy, Prince Sattam Bin Abdul Aziz University, Al Karj, SAU; 5 Department of Physical Therapy, Jazan University, Jazan, SAU; 6 Department of Physical Therapy and Health Rehabilitation, Majmaah University, Majmaah, SAU; 7 Department of Physical Therapy and Health Rehabilitation, Majmaah University, Riyadh, SAU

This article has been corrected at the request of the authors to change the affiliations as follows:

Abdullah O. Alotaibi Original: Department of Nursing, Majmaah University, Riyadh, SAU Department of Biomedicine and Prevention, University of Rome Tor Vergata, Rome, ITA Corrected: Department of Nursing, College of Applied Medical Science, Majmaah University, Almajmaah, SAU. Nursing Sciences and Public Health, Department of Biomedicine and Prevention, University of Rome Tor Vergata, Rome, ITA

Wdad Alanazy Original: Department of Nursing, Majmaah University, Riyadh, SAU Corrected: Department of Nursing, College of Applied Medical Science, Majmaah University, Almajmaah, Saudi Arabia

Jazi S Alotaibi Original: Department of Nursing, Majmaah University, Riyadh, SAU Corrected: Department of Nursing, College of Applied Medical Science, Majmaah University, Almajmaah, Saudi Arabia

The authors deeply regret that these errors were not identified and addressed prior to publication.

 

